# Long-Term Survival in Metastatic Pancreatic Adenocarcinoma of Intestinal Type

**DOI:** 10.3390/jcm13175034

**Published:** 2024-08-25

**Authors:** Gabriela Rahnea-Nita, Laura-Florentina Rebegea, Valentin Titus Grigorean, Ionuţ Simion Coman, Violeta Elena Coman, Iancu Emil Pleşea, Anwar Erchid, Costin George Florea, Mircea Liţescu, Roxana-Andreea Rahnea-Nita

**Affiliations:** 1 Department of Oncology-Palliative Care, “Sf. Luca” Chronic Diseases Hospital, 041915 Bucharest, Romania; gabriela.rahnea-nita@umfcd.ro (G.R.-N.); roxana.rahnea-nita@umfcd.ro (R.-A.R.-N.); 2 Specific Disciplines Department, Faculty of Nursing and Midwifery, “Carol Davila” University of Medicine and Pharmacy, 020021 Bucharest, Romania; 3 Medical Clinical Department, Faculty of Medicine and Pharmacy, “Dunărea de Jos” University, 800008 Galaţi, Romania; laura.rebegea@ugal.ro; 4 Department of Radiotherapy, “Sf. Apostol Andrei” Hospital, 800578 Galaţi, Romania; 5 10th Clinical Department—General Surgery, Faculty of Medicine, “Carol Davila” University of Medicine and Pharmacy, 020021 Bucharest, Romania; ionut.coman@umfcd.ro (I.S.C.); elena.coman@umfcd.ro (V.E.C.); 6 Department of General Surgery, “Bagdasar-Arseni” Clinical Emergency Hospital, 041915 Bucharest, Romania; erchid.anwar@yahoo.com (A.E.); costinflorea1990@gmail.com (C.G.F.); 7 Department of Histopathology, “Bagdasar-Arseni” Clinical Emergency Hospital, 041915 Bucharest, Romania; pie1956@yahoo.com; 8 Discipline of Surgery and General Anesthesia—“Sf. Ioan” Clinical Emergency Hospital, 2nd Department, Faculty of Dental Medicine, “Carol Davila” University of Medicine and Pharmacy, 020021 Bucharest, Romania; mircea.litescu@umfcd.ro; 9 Department of General Surgery, “Sf. Ioan” Clinical Emergency Hospital, 042122 Bucharest, Romania; 10 8th Clinical Department—Radiology, Oncology and Hematology, Faculty of Medicine, “Carol Davila” University of Medicine and Phamacy, 020021 Bucharest, Romania

**Keywords:** metastatic pancreatic cancer, long-term survival, chemotherapy

## Abstract

**Introduction and Literature Review:** Pancreatic cancer is often diagnosed in an advanced/metastatic stage, as it is a very aggressive type of cancer. The prognosis of pancreatic cancer is extremely unfavorable. The mean survival rate for patients with metastatic pancreatic adenocarcinoma is 3–6 months. Stage IV pancreatic cancer has a five-year survival rate of 1.3% to 13%. This article presents recent data regarding the oncologic management of metastatic pancreatic cancer. **Case presentation:** We present the case of a female patient who was 49 years old at the time of diagnosis, in June 2021. The patient was diagnosed with stage IV pancreatic neoplasm (due to liver metastases). The diagnosis was made by histopathological and immunohistochemical examination, which corroborated imaging investigations. The patient underwent four lines of chemotherapy between July 2021 and July 2024, undergoing partial response to the disease. The patient is a long-term survivor of metastatic pancreatic cancer (3 years in July 2024). **Discussions:** the peculiarity of this case is long-term survival (3 years and a month at the date when this article is being written) in a patient with pancreatic cancer and liver metastases. **Conclusions:** histopathological type, good performance status, CEA, and CA tumor markers 19.9 within normal limits may be favorable prognostic factors for long-term survival in metastatic pancreatic carcinoma.

## 1. Introduction

Pancreatic cancer is a very aggressive type of cancer, and it has an extremely unfavorable prognosis, as it is often diagnosed in an advanced/metastatic stage [1,2].

In the USA, the overall pancreatic cancer incidence per 100,000 individuals was 13.0 during the years 2009–2018 [3].

In Europe, the incidence of pancreatic cancer per 100,000 individuals was 7.6 in men and 4.9 in women [4].

Pancreatic cancer is the fourth leading cause of death due to cancer worldwide [2]. The mean survival rate for patients with metastatic pancreatic adenocarcinoma is 3–6 months [5].

The incidence and mortality are higher in men than in women.

Pancreatic ductal adenocarcinoma with pancreatobiliary-type differentiation is the most common and frequent type of pancreatic cancer, occurring in more than 85% of pancreatic cancer cases.

Adenocarcinoma of intestinal-type differentiation is a rare form, with a similar morphology to intestinal metaplasia, which occurs in various anatomical sites. It has been recognized in the last 16 years as a variant that can occur in the pancreas [6,7,8,9].

Regarding immunohistochemical tests, CDX 2 is a positive gene both in colorectal carcinoma and pancreatic adenocarcinoma with intestinal-type differentiation. The CDX 2 gene is negative in pancreatic adenocarcinoma with pancreatobiliary-type differentiation [6,10,11]. Colorectal carcinomas expressed aberrant immune profiles of CK20/CK7 [12]. In ampullary carcinomas, immunoreactivity against CK20 and MUC1 is used in histologic examination for determining the histotype but without prognostic significance [13].

Long-term survival among patients with pancreatic adenocarcinoma is very rare. Stage IV pancreatic cancer has a five-year survival rate of 1.3% [14]. Very few patients who did not undergo surgery but only oncological treatment will survive more than 5 years [15].

The intestinal type has a better prognosis than the pancreaticobiliary histological type of differentiation [11,16].

Chemotherapy offers benefits regarding the survival of patients with advanced pancreatic cancer [17,18,19].

### Literature Review

We reviewed the most relevant articles recently published, and we selected 11 studies with clinical impact, published in the years 2023 and 2024.

The main updates include epidemiology, multiagent chemotherapy, and predictors of long-term survival.

These selected articles are synthesized and listed in Table 1.

In a literature review conducted in 2024, Hernández-Blanquisett et al. highlighted the risk factors of pancreatic cancer, i.e., genetic disorders (familial pancreatic cancer, Lynch syndrome, Peutz–Jeghers syndrome), diabetes, obesity, pancreatitis, smoking, and alcohol consumption.

The lack of symptoms at the onset of the disease leads to a late diagnosis, when the disease has reached an advanced stage in which no resection is possible, due to locally advanced involvement or distance metastases.

Systemic chemotherapy is the best therapeutic alternative for metastatic pancreatic cancer, which increases survival and the quality of life [4].

The first-line treatment is single-agent chemotherapy gemcitabine, FOLFIRINOX combination regimen, and gemcitabine plus capecitabine. Another first-line regimen is first-line cisplatin, carboplatin, or oxaliplatin plus/minus olaparib.

Regarding the second-line treatment, after gemcitabine failure, the therapeutic regimens include oxaliplatin, folinic acid, and 5-FU (OFF), or 5-FU/LV (FF) or nanoliposomal irinotecan or nanoliposomal irinotecan + FF.

Although first- and second-line treatments have not significantly changed in the past years, a new therapeutic alternative, i.e., irinotecan liposomal, has proven to increase the survival rate [2,20].

In a retrospective study on 72 patients for the third-line treatment conducted between June 2013 and January 2023 by Lu et al., 16 patients underwent chemotherapy combined with targeted therapy or immunotherapy, and 36 patients underwent chemotherapy alone. Their study revealed that the third-line treatment can prolong the survival rate among patients with metastatic pancreatic carcinoma. In this study, targeted therapy or immunotherapy failed to improve survival benefits compared to chemotherapy [21].

A review performed by Balsano et al. in 2023 regarding immune checkpoint inhibitors alone or in combination with chemotherapy, or targeted therapies, revealed that these treatments have not changed the clinical results in pancreatic cancer. Moreover, it is said that there are high expectations of PARP-inhibitor combinations, CAR-T-cell therapy, and vaccines [22].

A study conducted between 2006 and 2017 by Valdera et al. about non-metastatic pancreatic cancer patients undergoing operation revealed that pre-/postoperative poly-chemotherapy can improve the 5-year overall survival compared to single-agent chemotherapy [23].

In an overview published in 2023, Garajová et al. analyzed two therapeutic lines: The first line consisted of two regimens, namely FOLFIRINOX followed by maintenance therapy after first-line treatment with FOLFIRI or 5-FU and gemcitabine/nab-paclitaxel, followed by maintenance therapy with gemcitabine. It was mentioned that there was no standard second-line treatment. Moreover, there was also a reference to rarer histological types, namely those with BRCA mutations, or adenosquamous carcinoma, pancreatic acinar cell carcinoma, and pancreatic mutations [24].

In 2023, Fronk et al. published the first case report in the literature of a patient with an ovarian tumor, which proved to be a metastasis of pancreatic adenocarcinoma with intestinal-type differentiation. The authors revealed that pancreatic cancer with intestinal-type differentiation is frequently cytokeratin CK7-positive and CK20-positive, while that with pancreaticobiliary-type differentiation is frequently CK7-positive and CK20-negative [6].

Distinguishing intestinal tumors from pancreaticobiliary tumors is an important prognostic factor [25].

The expression of mucin proteins MUC1 and/or MUC4 is an independent prognostic factor among patients with pancreatobiliary differentiated adenocarcinomas, considering that these patients have an unfavorable prognosis. Moreover, the CK7 expression, observed in pancreatobiliary differentiated adenocarcinomas, is an unfavorable prognostic factor. By comparison, the expression of CK20, MUC2, and CDX2 identifies tumors with intestinal differentiation, which have a good prognosis [26].

**Table 1:** Comparative analytical data.

No.	Author—Year	Subject	Reference No.
1	Hernández-Blanquisett—2024	Review	[2]
2	Fronk—2023	Case Report	[6]
3	Stoffel—2023	Epidemiology	[20]
4	Lu—2023	Third-line treatment	[21]
5	Balsano—2023	Immunotherapy	[22]
6	Valdera—2024	Multi-agent chemotherapy	[23]
7	Garajová—2023	Overview	[24]
8	Allard—2023	Adenocarcinoma ampulla	[25]
9	Zhu X—2023	Level of CA 19.9	[27]
10	Xue K—2023	Neo-adjuvant therapy	[28]
11	Javed A.A.—2024	Predictors of long-term survival	[29]

In a study published in 2023, Xinzhe Zhu et al. revealed the fact that stage IV pancreatic cancer patients with a normal level of carbohydrate antigen 19-9 (CA19-9), the best-validated biomarker for pancreatic cancer, have a 5-year survival rate higher than patients with stage I–IV who have a more increased level of CA marker 19.9 (22.4% vs. 6.8%). The conclusion of the study was that there is a subgroup of pancreatic cancer patients with a normal level of CA marker 19.9, with low glucose and high insulin [27].

An analysis conducted by Kang Xue et al. revealed that neo-adjuvant therapy followed by pancreatectomy with major arterial resection increases the survival rate of patients with unresectable pancreatic cancer involving the arteries [28].

In an analysis conducted by Ammar A. Javed et al. regarding the prognostic factors for long-term survival in operated pancreatic cancer patients, the following were identified as predictive factors for long-term survival: gender, body mass index, preoperative levels of CA 19.9, CEA, and albumin, pathologic T-stage, lymphovascular and perineural invasion, tumor stage, lymph node disease, metastatic disease, adjuvant therapy, vascular resection, operative blood loss, and perioperative blood transfusion [29].

Tumor grade, TNM staging, vascular invasion, the neutrophil–lymphocyte ratio, and the platelet–lymphocyte ratio are also independent risk factors in pancreatic cancer patients [30,31].

Further studies are necessary to determine the best approach for patients with metastatic pancreatic cancer.

## 2. Case Presentation

We present the case of a 49-year-old female patient with metastatic pancreatic cancer T4N0M1 who presented to “Bagdasar-Arseni” Emergency Clinical Hospital in Bucharest in June 2021 for epigastric pain radiating in the right hypochondrium. The symptoms occurred a year before, and they exacerbated in the last two months.

The patient’s characteristics are presented in Table 2.

The clinical examination revealed a thin abdomen, no signs of peritoneal irritation, palpable upon palpation at the level of the right hypochondria, and biologically within normal limits.

The upper digestive endoscopy revealed the esophagus with a hiatal hernia, the stomach with a moderate amount of bile fluid, edematous hyperemic mucosa, normal pylorus, normal duodenum, and medulla oblongata.

The abdominal ultrasound revealed a non-homogenous liver, multiple hyperechogenous images, and no dilated bile ducts. Cholecystectomy revealed normal bile ducts and the portal vein within normal limits. The pancreas was not visible. The spleen and both kidneys were normal.

The computed tomography of the thorax, abdomen, and pelvis performed in June 2021 revealed normal pulmonary and mediastinal features. The tumor mass was located in the uncinated process with a polylobate shape and dimensions of 47/32/42 mm. The lesion was spontaneously hypodense, it loaded non-homogenously after contrast medium injection and infiltrated duodenum III and the lower knee, as well as the adjacent fat.

**Figure 1:**
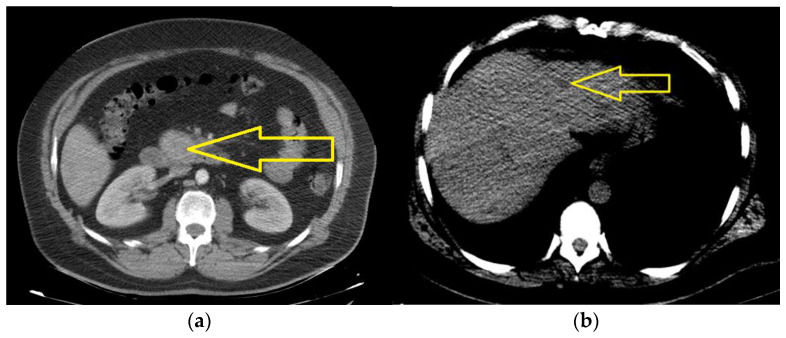
The computed tomography of the abdomen with intravenous contrast medium (June 2021): (**a**) a tumor mass located in the uncinated process, with a polylobate shape and dimension of 47/32/42 mm (yellow arrow); (**b**) secondary hepatic determinations with dimensions of up to 12 mm (yellow arrow).

The liver was normal in dimension, presenting multiple nodular lesions with dimensions of up to 12 mm, with secondary hepatic determinations. The gallbladder was surgically removed. Juxtacentrimetic osteocondensed lesions were located in the vertebral body and the right pedicle L3 (Figure 1a,b).

Surgery was performed in June 2024, and multiple secondary hepatic determinations were revealed in both hepatic lobes, a tumor formation in the body and the uncinated process of the pancreas, with the invasion of the mesenteric vessels and duodenum III, with slight ischemia of the small intestinal loops. Precolic gastrojejunostomy with Braun enteroenterostomy as well as a biopsy of the hepatic metastasis were performed, along with the histopathological examination. The postoperative evolution was favorable.

The histopathological examination report in June 2021 was as follows: location—hepatic metastasis; hepatic tissue with microsteatosis containing tumor carcinomatous nodules distributed under the form of acini and trabeculae. The case required the immunohistochemical analysis of the piece to determine the histogenesis.

The immunohistochemical analysis in July 2021 revealed the following:

- CK7—negative in tumor cells; positive internal control;
- CK20—negative in tumor cells;
- GATA3—negative in tumor cells;
- PAX8—slightly positive nuclear focus in the tumor cells (aberrant and uninterpretable immunohistochemical expression);
- CDX2—highly nuclear positive in more than 95% of the tumor cells.

The expression pattern indicated the diagnosis of NOS intestinal adenocarcinoma. Conclusion was NOS intestinal adenocarcinoma.

The patient presented to the Oncology–Palliative Care Department of “St. Luca” Chronic Disease Hospital in Bucharest, in July 2021, with an Eastern Cooperative Oncology Group (ECOG) Performance Status = 1, with the diagnosis of pancreatic tumor invading duodenum III and the great vessels (the mesenteric artery and vein). Secondary hepatic and vertebral determinations were identified.

According to the recommendation of the Multidisciplinary Oncological Board, in July 2021, the first-line regimen of chemotherapy FOLFOX 4 was initiated. This treatment was administered between July and December 2021. The chemotherapy regimen was oxaliplatin (85 mg/m^2^ on day 1), leucovorin (LV) (200 mg/m^2^ on days 1 and 2), and 5-fluorouracil (400 mg/m^2^ as a bolus and 600 mg/m^2^ as a 22 h infusion on days 1 and 2) every 2 weeks. The treatment was well tolerated under antiemetic therapy (palonostron 250 micrograms, intravenously about 30 min before starting chemotherapy).

An upper digestive endoscopy was performed in November 2021, as well as a lower digestive endoscopy, and the results revealed the following:

The upper digestive endoscopy: esophagus—normal aspect of the mucosa; fluid leaks upon withdrawal: the Z line at 33 cm from the AD; the stomach—HGTH of approximately 3 cm; antral hyperemic mucosa: prepyloric eroded papule; significant bile pyloric reflux: permeable; medulla oblongata: normal aspect of the mucosa; duodenum II—normal aspect of the mucosa; biopsy of the gastric antrum for Helicobacter Pylori—positive result. The following treatment was recommended: proton pump inhibitors (pantoprazole 40 mg daily) + antibiotics (clarithromycin 500 mg + amoxicillin 1000 mg twice daily), for 7 days.

Lower digestive endoscopy revealed rectum, sigmoid colon, descending, transverse and ascending colon, cecum with normal endoscopic appearance, and thin ileocecal valve.

**Figure 2:**
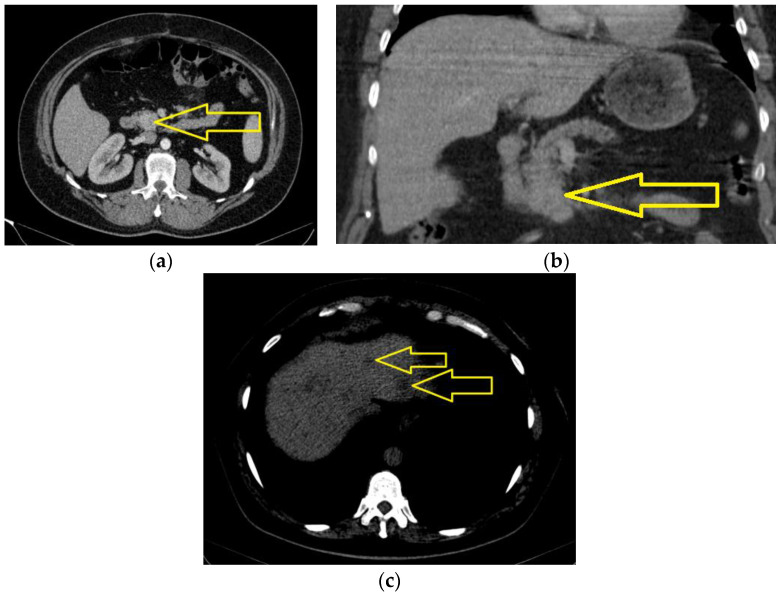
The computed tomography of the abdomen with intravenous contrast medium (January 2022): a polylobate expansive process of approximately 40/30/28 mm, located in the uncinated process and partially in the pancreatic cephalic area infiltrating duodenum III and coming in contact with the superior mesenteric vein: axial section (**a**) and coronal section (**b**) (yellow arrows); (**c**) secondary hepatic determination, the largest with the diameter of approximately 14 mm (yellow arrows).

Computed tomography of the head, thorax, abdomen, and pelvis was performed in January 2022, revealing a polylobate expansive process of approximately 40/30/28 mm, located in the uncinated process and partially in the pancreatic cephalic area. The described mass was iodophilic, non-homogenous, infiltrating duodenum III, and coming in contact with the superior mesenteric vein. The rest of the pancreas was atrophic, without dilations of the Wirsung duct. Adenopathy was detected in the mesenteric fact with axial dimensions of approximately 17/15 mm. The CBP had a maximum diameter of roughly 10 mm in the hilum. The liver had a non-homogenous structure due to the presence of spontaneously hypodense focal lesions disseminated in both hepatic lobes, visible especially on the CT without contrast medium, and the largest of them had a maximum diameter of approximately 14 mm, secondary hepatic determinations that were already diagnosed. Infracentrimetric osteosclerotic lesions were located in the vertebral body L3, S1, and at the level of the right femoral neck (Figure 2a–c).

In January 2022, after the re-assessment of the patient, the Multidisciplinary Oncological Board recommended changing the treatment line to second-line chemotherapy with gemcitabine plus carboplatin, a treatment that the patient underwent between January 2022 and October 2022. The chemotherapy regimen was gemcitabine 1000 mg/m^2^ on day 1 and carboplatin at an AUC of 4 of a 3-week cycle. The treatment was well tolerated under antiemetic therapy (palonosetron).

In April 2022, a bone scan was performed, indicating minimal non-specific L4 and bilateral femoral capture anomalies. It required monitoring and correlation with CT and MRI imaging.

A new CT of the head, thorax, abdomen, and pelvis was performed in October 2022, revealing the following: a polylobate expansive process of approximately 25/27 mm axial compared to the 40/30 mm in the previous examination, located in the uncinated process and partially in the pancreatic cephalic area, with smoother outlines and less contrast intake; it infiltrated duodenum III and came in contact with the superior mesenteric vein with a thin fatty lining at the time.

The rest of the pancreas was slightly atrophic, without dilations of the Wirsung duct.

Adenopathy was detected within the mesenteric fat with axial dimensions of approximately 13/13 mm and denser mesenteric fat; for secondary hepatic determinations, progression was observed in number and dimensions.

The Multidisciplinary Oncological Board gathered in October 2022 recommended a third-line treatment, namely irinotecanum (irinotecan pegylated liposomal) along with calcium folinate (LV) plus fluorouracil, a treatment that the patient underwent between November 2022 and May 2023. The chemotherapy regimen was irinotecan pegylated liposomal 70 mg/m^2^, intravenously, followed by LV 400 mg/m^2^ intravenously, followed by 5-FU 2400 mg/m^2^, intravenously over 46 h, administered every 2 weeks. The side effects of pegylated liposomal irinotecan were mild nausea and vomiting; antiemetic therapy was administrated (palonostron).

**Figure 3:**
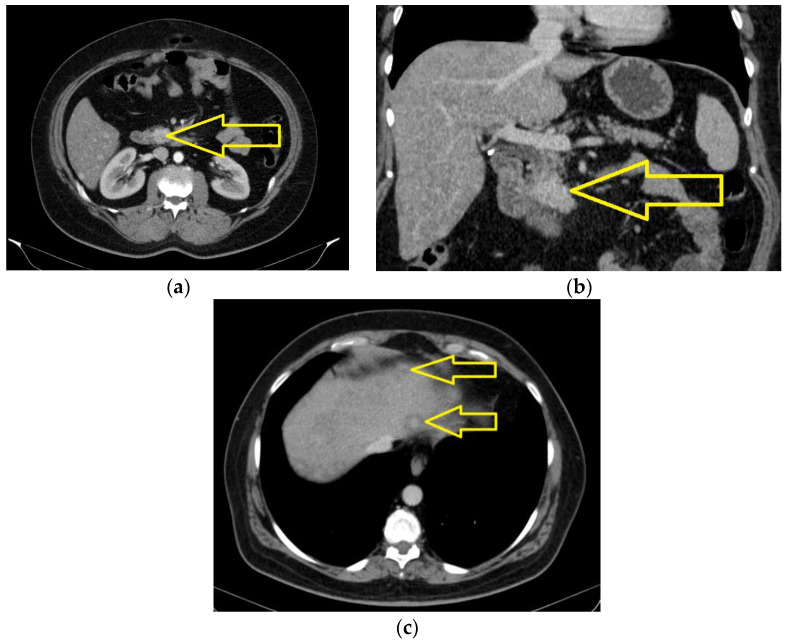
The computed tomography of the abdomen with intravenous contrast medium (May 2023). An expansive polylobate process of approximately 21.4/27 compared to 25/27 mm axially at the previous examination: axial section (**a**) and coronal section (**b**) (yellow arrows); (**c**) liver of increased dimensions (the cranial–caudal diameter of the LDH is 17.3 cm), spontaneously hypodense focal lesions disseminated in both hepatic lobes; some of them with a central contract uptake, the largest of them located in the IV B segment, with a maximum diameter of approximately 20/14 mm—known secondary hepatic determination in numerical progression (yellow arrows).

The computed tomography of the head, thorax, abdomen, and pelvis was performed in May 2023 and revealed an expansive polylobate process of approximately 21.4/27 compared to the 25/27 mm axially at the previous examination, located in the uncinated process and partially in the pancreatic cephalic area, with smoother outlines and less contrast uptake; it infiltrated duodenum III and came in contact with the superior mesenteric vein, with a thin fatty line of demarcation at this point. The rest of the pancreas was slightly atrophic, without dilations of the Wirsung duct. Adenopathy was detected in the mesenteric fat with axial dimensions of approximately 13/13 mm, as well as densification of the mesenteric fat. Pericephalic, pancreatic, and celio-mesenteric lymph nodes were observed with a short infra-juxacentimetric axis. Liver of increased dimensions (cranial–caudal diameter of LDH 17.3 cm), clear, and regular outline was observed, with non-homogenous structure due to the presence of spontaneously hypodense focal lesions disseminated in both hepatic lobes; some of them with a central contract uptake, the largest being located in the IV B segment, with a maximum diameter of approximately 20/14 mm—known secondary hepatic determinations in numerical progression (Figure 3a–c).

The Multidisciplinary Oncological Board gathered in May 2023 recommended the fourth-line treatment with gemcitabine plus capecitabine, a treatment that the patient underwent between June 2023 and July 2024 (the date when this article was written). The chemotherapy regimen was gemcitabine 1000 mg/m^2^ by 30 min infusion on day 1 plus oral capecitabine 650 mg/m^2^ twice daily on days 1 to 14, every 3 weeks. The treatment was well tolerated under antiemetic therapy (palonosetron).

In September 2023, a bone scan was performed. The bone scan was not suggestive of the occurrence of secondary bone determinations in the skeleton.

In January 2024, a new CT scan of the head, thorax, abdomen, and pelvis was performed, revealing the following: The expansive process with a polylobate appearance was maintained, with dimensions of approximately 26/12 mm, located in the uncinated process and partially in the cephalic pancreatic area (a reduction in dimension from the previous examination); it infiltrated duodenum III and came in direct contact with the superior mesenteric vein with which it maintained the demarcation limit.

The rest of the pancreas was slightly atrophic, without dilations of the Wirsung duct.

Adenopathy was detected in the mesenteric fat with axial dimensions of approximately 13/10 mm, as well as densification of the mesenteric fat. The pericephalic pancreatic and celio-mesenteric lymph nodes were observed with a short infra-juxtacentimetric axis.

**Figure 4:**
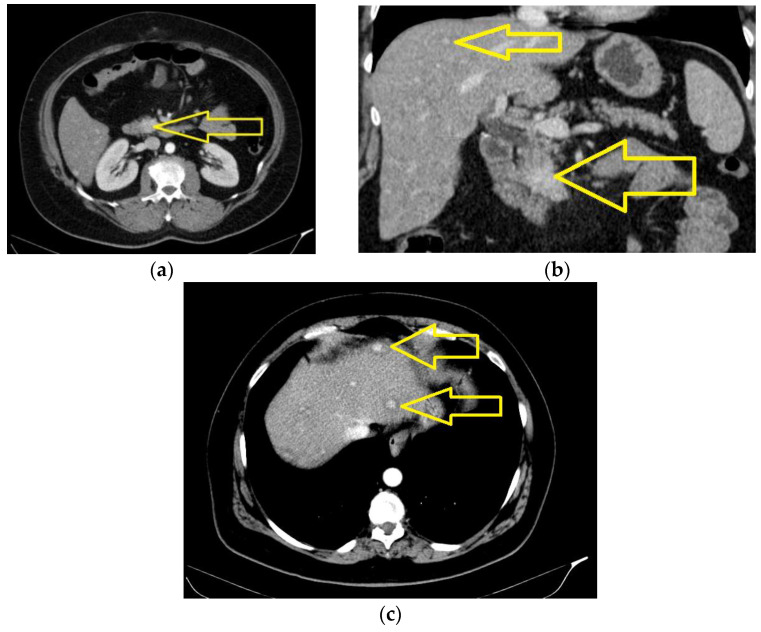
The computed tomography of the abdomen with intravenous contrast medium (January 2024): an expansive process with a polylobate aspect, with dimensions of approximately 16/12 mm, located in the uncinated process and partially in the cephalic pancreatic area (reduction in dimensions compared to the previous examination): axial section (**a**) and coronal section (**b**) (yellow arrows); (**c**) known secondary hepatic determination in apparently numerical regression (yellow arrows).

Liver of increased dimensions (the cranial–caudal diameter of the LDH is 17.3 cm), clear, and regular outline was observed, with a non-homogenous structure due to the occurrence of spontaneously hypodense focal lesions disseminated in both hepatic lobes, some of them with central contract uptake; the largest of them was located in the fourth B segment, with a maximum diameter of approximately 20/14 mm—known secondary hepatic determination in apparently numerical regression (the lesions in segments VII and VIII were no longer individualized with certainty) (Figure 4a–c).

In April 2024, a lumbosacral MRI was performed, and no secondary bone determinations were identified.

In July 2024, the general state of the patient was very good, with a performance status ECOG = 1, with no symptoms, under treatment with gemcitabine plus capecitabine; the next re-assessment will be in August 2024. The tumor markers carcinoembryonic antigen (CEA) and carbohydrate antigen (CA 19.9) and the regular laboratory tests were within normal limits throughout the entire period, between June 2021 and July 2024.

The values of tumor markers CEA and CA19.9 are presented in Table 3.

## 3. Discussion

The pancreas is made up of three parts, namely the head of the pancreas (the bulkiest part, surrounded by the duodenum; under the head of the pancreas, there is a small prominence called the uncinate process), the body of the pancreas (the extended portion of the pancreas, positioned perpendicularly on the vertical axis of the pancreas), and the tail of the pancreas (the terminal portion of the pancreas, located between the spleen and the left kidney).

The pancreas produces two types of secretions: exocrine (pancreatic juice from the acinar cells), which enters the duodenum through the main and accessory pancreatic ducts, and endocrine (glucagon and insulin from the pancreatic islets of Langerhans), which enters the blood [32].

In 2007, Jorge Albores-Saavedra et al. published a study in which they reported the results of 11 patients with ductal carcinomas of the pancreas that were morphologically similar to colon adenocarcinomas (ductal adenocarcinomas with intestinal phenotype). Six of the patients were female, and five were male. These pancreatic carcinomas of the intestinal type represented 10% of the 110 operated pancreatic cancers. All 11 cases had their origin in the head of the pancreas, and 5 of them presented ganglionic metastases at the time of surgery. Only one patient survived for 5 years [33].

This paper presents the case of a female patient from a rural area, aged 49 at the time of diagnosis, with pancreatic cancer and multiple hepatic metastases. The patient is a non-smoker, does not consume alcohol, has obesity class I, and likes sweets very much. She has always been a housewife. Currently, she has a disability pension. She presented to the Oncology–Palliative Care Department of “St. Luca” Chronic Disease Hospital in Bucharest, in July 2021, during the alert state of the COVID-19 pandemic [34]. At the time of presentation, she had a performance status ECOG = 1, which remained unchanged throughout the study period, until July 2024. She is an optimistic person and very confident in the medical team, respecting all the medical recommendations.

In the case of the patient in our study, the diagnosis was made through the biopsy of hepatic metastasis, and the conclusion of the histochemical examination was NOS adenocarcinoma of the intestinal type. Taking into account the fact that computed tomography revealed a tumor located in the uncinated process and partially in the cephalic pancreatic area, infiltrating duodenum III and in direct contact with the superior mesenteric vein, as well as secondary hepatic determinations, corroborating with the immunohistochemical results, the diagnosis was pancreatic neoplasm stage IV and hepatic metastases (adenocarcinoma of intestinal type).

The patient underwent systemic chemotherapy, according to the recommendation of the Multidisciplinary Oncological Board. The first therapeutic line was the FOLFOX 4 regimen, administered for 8 months and under which a slight reduction in dimensions of the pancreatic tumor was possible but with a slight increase in dimensions of the hepatic metastases.

The second-line treatment was the regimen with gemcitabine plus carboplatin, administered for 10 months, followed by a reduction in the dimension of the uncinated process, from 40/30 mm to 25/27 axially and a numerical progression of secondary hepatic determinations.

The third-line treatment was the regimen with irinotecanum (irinotecan pegylated liposomal) along with calcium folinate plus fluorouracil, a treatment undergone for 7 months by the patient, followed by a numerical progression of the secondary hepatic determinations.

The fourth-line treatment was a regimen with gemcitabine plus capecitabin, a treatment undergone by the patient for a year and a month. Upon the assessment performed at 7 months from the beginning of this treatment, a numerical regression of the hepatic metastases was observed [21,23,24,35].

Also, we would like to mention that our patient tested positive for Helicobacter Pylori, for which she underwent antibiotic treatment.

Recent studies have highlighted that the use of antibiotics in patients with metastatic pancreatic cancer may predict better treatment results through the antibiotic-associated modulation of the microbiome. In a retrospective study published in 2021, Mohindroo C. et al. evaluated clinical data of patients with resectable or metastatic pancreatic ductal adenocarcinoma, in the period 2003–2017 and highlighted that the use of antibiotics is associated with an increased progression-free and overall survival, especially in patients who received concomitant gemcitabine-based chemotherapy [36].

In a retrospective cohort study of 3850 older adults with metastatic pancreatic ductal adenocarcinoma, published in 2023, Fulop DJ et al. analyzed data for patients diagnosed between 2007 and 2017 and found that perichemotherapy antibiotics were associated with improved overall survival among patients treated with first-line gemcitabine but not fluorouracil [37].

Prospective studies are needed to evaluate the effect of antibiotic administration associated with chemotherapy on survival in metastatic pancreatic cancer.

The peculiarities of this case are as follows:

- The histopathological type: the adenocarcinoma of the intestinal type;
- The corroboration of the immunohistochemical data with the imaging data in order to make the diagnosis;
- The maintenance of the performance status ECOG = 1 throughout the entire period;
- The long-term survival (3 years and a month at the date when the article was written) in a patient with pancreatic cancer and hepatic metastases who underwent more lines of chemotherapy;
- The tumor markers CEA and CA 19.9 with normal values throughout the entire period of 3 years.

## 4. Conclusions

The results of this study reveal that histopathological type (adenocarcinoma of the intestinal type), good performance status (ECOG = 1), tumor markers CEA and CA 19.9 within normal limits, young age (49 years), and female gender may be favorable prognostic factors for long-term survival in metastatic pancreatic carcinoma. Moreover, the change in therapeutic lines contributes to increasing the overall survival rate.

Further studies are necessary considering the complex approach of patients with metastatic pancreatic cancer, in order to improve their survival rate and their quality of life.

## Figures and Tables

**Table 2:** Patient’s characteristics.

Age	49
Gender	Female
Life environment	Rural
Studies	Elementary school
Type of hospitalization	Continuous care
Profession	Housewife
Smoking	No
Alcohol	No
Eating habits	Sweets
Other diseases	Obesity Class I
Family Antecedents	No
ECOG Performance status	1
Weight	75 kg
Height	155 cm
Body Surface	1.74 m^2^
Body Mass Index (BMI)	31.22
Blood markers (before the treatment-2021)	Hemoglobin = 15.8 mg/dL, leukocyte = 12.2 × 10^3^/µL sange platelets = 195 × 10^3^/µL, glucose = 84 mg/dL, creatinine = 0.9 mg/dL, total bilirubine = 0.49 mg/dL, alanine transaminase (ALT) = 36 U/L aspartate transaminase (AST) = 31 U/L, gamaglutamiltranspeptidase (GGT) = 57 U/L, alkaline phosphatase = 249 U/L

**Table 3:** Tumor markers CEA and CA19.9.

Nr.crt	Tumor Markes	July 2021	January 2022	December 2022	May 2023	July 2024	Normal Values
1	CEA	0.54	0.72	0.93	0.83	0.79	0–5.093 ng/mL
2	CA 19.9	5.48	2.5	10.01	10.9	25.6	2–37 U/mL

## Data Availability

Data can be obtained upon contacting the first author.
